# Bilateral Cerebral Hemorrhagic Infarctions: An Early Presentation of Carbon Monoxide Poisoning

**DOI:** 10.7759/cureus.17772

**Published:** 2021-09-06

**Authors:** Raima Kaleemi, Shayan S Anwar, Anwar Ahmed

**Affiliations:** 1 Department of Radiology, Aga Khan University Hospital, Karachi, PAK

**Keywords:** carbon monoxide poisoning, hemorrhagic infarcts, hypoxic injury, globus pallidus, magnetic resonance imaging, white matter demyelination

## Abstract

Carbon monoxide (CO) poisoning is one of the most common causes of morbidity secondary to accidental or intentional exposure. It is a potentially life-threatening disease. We present the case of a 23-year-old male patient who slept with a gas generator the whole night in a closed room. The next morning the patient presented to emergency with altered mentation. His Glasgow Coma Scale score was 8/15 on arrival. The patient had cerebral hemorrhages on presentation with diffuse cerebral hypoxic injury and bilateral globus pallidus signals. Hemorrhagic infarction in the brain is a rare presentation of CO poisoning and even rarer as an early manifestation of this disease. We present a case of bilateral posterior cerebral hemorrhagic infarctions with a diffuse hypoxic insult as an early presentation of CO poisoning in a young male, which to our knowledge has rarely been reported. Early imaging and prompt medical attention can be life-saving.

## Introduction

Carbon monoxide (CO) poisoning is a common cause of morbidity secondary to accidental or intentional toxic exposure. CO poisoning is a life-threatening condition with long-term neurologic morbidity. It has early and late manifestations. Early imaging findings frequently include diffuse hypoxic-ischemic encephalopathy and focal cortical injury, necrosis of the globus pallidus, and injury to the basal ganglia, thalamus, brainstem, and cerebellum. Late imaging findings comprise diffuse brain atrophy and cerebral white matter demyelination [1]. Bilateral symmetrical necrosis of the globus pallidus is a pathognomonic feature [2,3]. Here, we describe a case of bilateral posterior cerebral hemorrhagic infarctions along with a diffuse hypoxic injury to the brain as an early presentation of CO poisoning, which is rarely reported in the literature.

## Case presentation

A 23-year-old male was found with an altered level of consciousness at home in the morning according to his family. On the previous night, he had slept with a gas heater switched on. He had slept in his room with the doors and windows closed. He was taken to the local hospital, where after initial treatment and unenhanced computed tomography (CT) of the head, he remained in the same condition. The family then brought the patient to the emergency department of our hospital after 15 days. On arrival, his Glasgow Coma Scale score was E3M5V1. Table 1 lists the tests done in the emergency department. Only the report of the CT of the head was available which revealed bilateral posterior cerebral hemorrhagic infarcts in the watershed territory. Films were not available for review.

**Table 1:** Laboratory investigations. BUN: blood urea nitrogen; INR: international normalized ratio; SARS-CoV-2: severe acute respiratory syndrome coronavirus 2

Tests	Values	Normal range
BUN	20 mg/dL	6-20 mg/dL
INR	1.3	0.9-1.2
Sodium	133 mEq/L	136-144 mmol/L
Potassium	4.4 mEq/L	3.5-5.1 mmol/L
Calcium	8.4 mg/dL	8.6-10.2 mg/dL
Albumin	3.0 g/dL	3.5-5.2 g/dL
Fasting glucose	141 mg/dL	65-100 mg/dL
Hemoglobin	12.4 g/dL	12.3-16.6 g/dL
Platelets	203 × 10^9^/L	154-433 × 10^9^/L
Neutrophils	87.3%	34.9-76.2%
SARS-CoV-2 rapid antigen	Negative	-
Urine DR	150/uL hemoglobin + *Candida*	-

**Figure 1:**
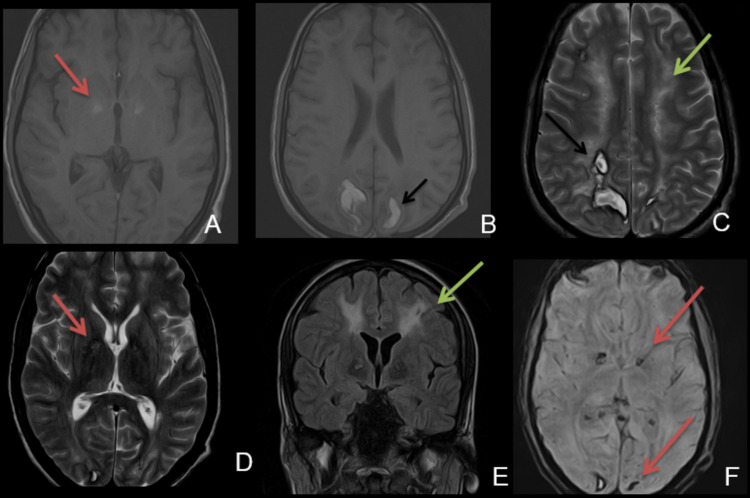
Multiplanar and multisequential MRI of the brain. A & B: T1 axial image showing T1 hyperintense signal in the globus pallidus (red arrow, typical finding of CO poisoning) and hemorrhage in the posterior parietal regions involving the gray-white matter junction and watershed areas (black arrow). C: T2 axial image showing abnormal T2 hyperintense signals in the subcortical white matter, centrum semiovale (green arrow), and hemorrhage in the posterior parietal regions (black arrow). D: T2 axial image showing T2 hyperintense signal in the globus pallidus (red arrow). E: FLAIR coronal image redemonstrating abnormal white matter signals. F: SWI showing signal dropout in the bilateral globus pallidus and posterior occipital region, more on the right (red arrow). CO: carbon monoxide; FLAIR: fluid-attenuated inversion recovery; MRI: magnetic resonance imaging; SWI: susceptibility-weighted imaging

Magnetic resonance imaging (MRI) and magnetic resonance venography of the brain were performed which showed abnormal signal intensity areas and almost symmetric involvement of bilateral cerebral hemispheres involving the posterior parietal and occipital lobes, which appeared hyperintense on T1/T2-weighted images with a peripheral rim of hypointensity. Corresponding areas showed signal dropouts on susceptibility-weighted imaging (SWI) sequence. No postcontrast meningeal or parenchymal enhancement was noted. No cerebral venous sinus thrombosis was found. Findings were representative of hemorrhagic infarctions (Figure 1, Panels B, C, F).

Bilateral symmetrical signals were seen in the corona radiata and periventricular regions, representing diffuse hypoxic white matter injury (Figure 1, Panels C, E). There were bilateral symmetrical abnormal signals in the globus pallidus on T1/T2-weighted images with signal dropouts on SWI, typical of CO poisoning (Figure 1, Panels A, D, F). There was no hydrocephalus or midline shift. Additionally, the corpus callosum, brainstem, and cerebellum were normal.

The patient had a clinical course of five days in the hospital. During his stay, he had continuous sinus tachycardia which was managed. He also developed acute kidney injury during his hospital stay, for which his medication was altered. Although he was advised to stay for further management, he left against medical advice.

## Discussion

CO is a colorless and odorless toxic gas. It is a byproduct of carbon-based compounds. It is the most lethal common poison worldwide resulting in neurological manifestations which are a frequent result of morbidity [4-6]. Patients with CO exposure present with various clinical signs and symptoms, of which altered mentation is a frequent presenting symptom [7].

The pathophysiologic mechanisms of CO poisoning are mainly divided into hypoxic and cellular [4,8]. The binding capacity of CO for heme is 250 times that of oxygen, causing tissue hypoxia in the acute setting. On the other hand, CO inhibits the mitochondrial electron transport enzyme system causing brain lipid peroxidation, and hence, manifesting delayed effects [4,9]. Brain injury secondary to CO poisoning usually involves bilateral globus pallidus, corpus callosum, thalamus, hippocampus, periventricular white matter, and cerebral cortex [10].

CO poisoning is a multisystem disorder and can be difficult to diagnose on time in the absence of proper history. Previously, CO poisoning has been associated with amnesia, encephalopathy, dysarthria, parkinsonism, peripheral neuropathy, cerebral hemorrhages, cardiotoxicity, and muscular necrosis [11].

CO poisoning can produce three different forms of symptoms: acute intoxication, recurrent symptom syndrome, and delayed neurocognitive or neuropsychiatric symptoms [11]. The interval between CO intoxication and MRI is important to determine. CO poisoning can be divided into four phases: the hyperacute phase within 24 hours; the acute phase within 1-7 days; the subacute phase within 8-21 days; and the chronic phase from 22 days onward [12]. Our patient presented in the subacute phase.

The most common lesions occur in the basal ganglia and cerebral gray and white matter [12]. Subcortical demyelination which is symmetric in nature is also a common finding accounting for delayed neurological symptoms. Focal hemorrhage in the basal ganglia is a common finding [13]. Cerebral edema can also lead to brain damage. Hemorrhagic infarcts have been rarely seen as an early manifestation of the condition [13,14]. The superiority of MRI imaging in diagnosing hemorrhagic infractions is well known; however, a CT scan is the modality of choice to exclude hemorrhage in the emergency setting [14].

Hemorrhagic infarctions in the brain due to CO poisoning is very rare. The possible mechanism can be microvascular impairment with a brain reperfusion injury initiated by free radicals. The microvascular injury produces ischemia and hemorrhage. Hemorrhage is further precipitated due to cardiovascular and hematological dysfunction by CO toxicity [12]. According to the history and imaging findings in our patient, it was logical to consider the case as a complication of prolonged CO poisoning.

In view of treatment options, such patients should promptly be removed from the primary source and given oxygen therapy, that is, 100% normobaric oxygen or hyperbaric oxygen. Patients should be evaluated for signs of cardiovascular and cerebrovascular ischemia and should be treated with oxygen to prevent long-term damage and delayed neurological sequelae [15].

## Conclusions

Hemorrhagic infarction in the brain is a rare presentation of CO poisoning and ever rarer as an early manifestation of the disease. Clinical history plays an important role in all scenarios and guides the physician in the right direction to manage such patients. The role of MRI is well known and early imaging to see the extent of the injury is crucial in management. Hence, we conclude that patients with CO poisoning can present with any of the described injuries to the brain in the literature, irrespective of the time since exposure.
